# Metabolic and Endocrine Insights in Donkeys

**DOI:** 10.3390/ani14040590

**Published:** 2024-02-10

**Authors:** Francisco J. Mendoza, Ramiro E. Toribio, Alejandro Perez-Ecija

**Affiliations:** 1 Department of Animal Medicine and Surgery, University of Cordoba, Cordoba 14014, Spain; alejandro.perez.ecija@uco.es; 2 Department of Veterinary Clinical Sciences, College of Veterinary Medicine, The Ohio State University, Columbus, OH 43210, USA; toribio.1@osu.edu

**Keywords:** asinine, Cushing, hyperinsulinemia, hyperlipemia, laminitis, obesity

## Abstract

**Simple Summary:**

Hyperlipemia, defined as abnormally high blood triglyceride concentrations, is the most common metabolic disease in donkeys. In contrast, metabolic syndrome, insulin dysregulation (ID) and pituitary pars intermedia dysfunction (PPID) are the most common endocrine disorders in this species. Although diagnostic approaches for these conditions are similar to horses, donkey-specific protocols must be used. Likewise, common drugs used to treat metabolic and endocrine disorders in horses are also used in donkeys, but there is minimal pharmacologic information (regimen dose, etc.) for most of them, except for pergolide.

**Abstract:**

Donkey medicine is gaining attention due to their increased use as companion animals, in shows, asinotherapy, etc. The increasing demand and unique aspects call for specialized care, requiring new information (physiology, infectious disorders, pharmacology, etc.). Since obesity is common in this species, hyperlipemia, metabolic syndrome and insulin dysregulation (ID) are common disorders in donkeys, in some cases with high mortality, either directly (multiorgan dysfunction) or indirectly due to poor quality of life (chronic laminitis). Donkeys have long-life expectancy and are often afflicted with pituitary pars intermedia dysfunction (PPID), a neurodegenerative and endocrine disease. Hyperlipemia is diagnosed based on high plasma triglyceride concentration in association with clinical findings and laboratory abnormalities from affected tissues (liver, kidney and pancreas). The measurement of resting serum insulin and plasma ACTH concentrations is the first step in ID and PPID diagnosis. In donkeys with clinical signs of ID (obesity or recurrent laminitis) or PPID (hypertrichosis, regional adiposity, laminitis and weight loss), where these hormones are in the normal or non-diagnostic range (donkey-specific cut-off values and reference ranges need to be established), dynamic tests are recommended (oral sugar test or thyrotropin-releasing hormone, respectively). Equine treatment protocols apply to donkeys, although pharmacological studies for most drugs, except pergolide, are lacking.

## 1. Introduction

Donkeys have a high energy efficiency and capacity to conserve water due to evolutionary adaptation to harsh and dry environments where food is scarce and/or of poor quality. Thus, when confined, fed hypercaloric and sugar-rich diets, and allowed access to pastures with high non-structural carbohydrate content, metabolic (obesity and hyperlipemia), endocrine (metabolic syndrome and insulin dysregulation) and clinical (laminitis) disturbances develop [1].

In addition, since donkeys are stoic and sturdy animals, with a long-life expectancy, neurodegenerative diseases of geriatric equids such as pituitary pars intermedia dysfunction (PPID; formerly Cushing’s disease) often develop. 

Considering that numerous differences have been demonstrated between donkeys and horses [2], and currently, more donkeys are attended by clinicians, dissemination of donkey-specific knowledge and approaches to different disorders could be valuable to improve care to avoid misdiagnosis, unnecessary treatments and additional costs.

## 2. Metabolic Diseases 

### 2.1. Hyperlipemia

#### 2.1.1. Introduction

Hyperlipemia is a dyslipidemia defined as abnormally high blood triglyceride concentrations (hypertriglyceridemia). It is one of the most common diseases in donkeys. Although epidemiologic data are lacking in this species, it is estimated to have a high incidence (10%) [3].
***Hyperlipemia keynotes***High incidence and mortality;More common in jennies, obese and small breeds;Glucagon could play a crucial role;Higher resting triglyceride and leptin than horses;Different lipid profile than horses;Non-specific clinical signs;Treatment similar to that for horses and ponies.

#### 2.1.2. Etiology

Hyperlipemia is caused by a negative energy balance from a primary clinical disorder (e.g., colic, pleuropneumonia, laminitis, parasitism, etc.), stressful situations (e.g., transport, husbandry changes, hospitalization, etc.) or physiological needs (e.g., pregnancy and lactation) [4].

#### 2.1.3. Epidemiology

Donkeys of any breed, age or gender can develop hyperlipemia; however, it is more frequent in smaller and miniature ones (10–20%) [4], where obesity is more common. Although gender is not a known predisposing factor, hyperlipemia tends to be more frequent and severe in jennies due to their higher tendency toward obesity [5], as well as pregnancy and lactation that can increase energy demands. It can be observed at any age, but it is more frequent in older animals, and is particularly linked to decreased insulin sensitivity [6]. However, it can also occur in newborn donkeys. Geography and economy are linked to obesity and the risk of hyperlipemia due to increased caloric intake and reduced physical activity. In certain latitudes where donkeys have access to high-quality pastures (e.g., the United States, Canada, Australia, Europe and some South American countries), obesity is frequent, but concurrent disease is the main risk factor for hyperlipemia (prevalence up to 72%) [3].

#### 2.1.4. Pathophysiology

Hyperlipemia in donkeys, contrary to its occurrence in species like cats, is not primary but a secondary disorder triggered by conditions leading to excessive lipolysis [4]. High levels of proinflammatory cytokines (TNF-α, IL-6), catecholamines, glucagon, glucocorticoids and adrenocorticotropic hormone (ACTH) as well as decreased insulin concentrations or sensitivity (insulin dysregulation) increase the activity of adipocyte hormone-sensitive lipase. This results in lipolysis with excessive mobilization of free fatty acids (FFA) that are re-esterified into triglycerides in the liver. The donkey liver is very efficient at transforming FFA into triglycerides. When this metabolic pathway is overwhelmed, triglycerides are released into the bloodstream as very low-density lipoproteins (VLDL). Lipoprotein lipase is an extracellular enzyme on the endothelial surface (predominantly present in adipose tissue and heart and skeletal muscle) that is important for triglyceride removal from the circulation and subsequent translocation into cells.

Glucagon is a lipolytic hormone with direct effects on hormone-sensitive lipase enzymatic activity, and it has been associated with hyperlipemia in donkeys. Feed-deprived obese donkeys had higher glucagon concentrations compared to non-obese feed-deprived donkeys and horses [7]. Obese donkeys also tend to have higher resting plasma glucagon concentrations than donkeys with a lower body condition score (BCS) [5]. In healthy donkeys and horses with similar plasma insulin concentrations, donkeys had higher plasma glucagon concentrations and glucagon-to-insulin molar ratios than horses [8]. Old donkeys have higher plasma glucagon concentrations compared to younger ones [5], which may contribute to the higher prevalence of hyperlipemia in older animals.

#### 2.1.5. Clinical Signs

Donkeys with hyperlipemia have non-specific clinical signs such as dullness, inappetence and behavioral changes, along with those related to the primary condition (colic, pleuropneumonia, laminitis, etc.) [9]. These signs can be exacerbated by organ failure due to fatty infiltration (liver disease, azotemia, dysrhythmias, etc.). 

#### 2.1.6. Diagnosis

The diagnosis of hyperlipemia is based on the measurement of blood triglyceride concentrations. This can be performed using validated point-of-care devices. It is important to note that healthy donkeys have higher (up to 150 mg/dL) resting triglyceride concentrations than horses [10]. The classification used in ponies and horses to differentiate between hypertriglyceridemia (blood triglycerides between 200 and 500 mg/dL), hyperlipidemia (blood triglycerides > 500–1000 mg/dL) and severe hyperlipidemia (blood triglycerides > 1000 mg/dL) has not been established in donkeys [4]. Considering that resting triglyceride concentrations are higher in donkeys and that they have a different lipid profile [11], this classification should be revised and adapted to donkeys.

Opalescent (lipemic) serum or plasma can interfere with the measurement of other blood analytes, such as creatinine, gamma-glutamyl transferase (GGT), phosphorus and calcium, causing a false decrease [12]. To obtain accurate analyte values, ultracentrifugation or extraction are recommended to clear the lipids and repeat the analysis. 

Other blood lipids can be measured, but the increase in plasma cholesterol and low-density lipoprotein (LDL) after fasting is slower compared to triglycerides, while minimal changes in plasma high-density lipoprotein (HDL) have been seen in some animals [7], despite HDL being the bulk of all lipoproteins in donkeys [11]. The increase in these blood lipids depends on obesity and endocrine status, with cholesterol being the most affected in obese donkeys [7]. The lipoprotein profile in donkeys differs to horses, with lower VLDL than horses [11]. 

Leptin and adiponectin are major energy-regulating hormones; however, they are rarely used in the clinical setting. Hyperleptinemia (>7.3 ng/mL) is better correlated with obesity than ID in horses [13]. In horses with ID, hyperleptinemia was associated with laminitis [14], but information in donkeys is lacking. Since healthy donkeys tend to have higher plasma resting leptin concentrations than horses [5], a cut-off specific to this species has to be generated. Hypoadiponectinemia (<8 µg/mL) has been proposed to have diagnostic value for obesity and to assess the risk of laminitis in horses [15], but it remains to be evaluated in donkeys. Given that glucagon plays a central role in fatty acid mobilization, it could be a potential biomarker for disease severity and for assessing the response to treatment, but it is not currently used in the clinical setting. C-peptide, a fragment of the proinsulin molecule that is released in equimolar amounts with insulin, is another factor that may have diagnostic and prognostic value for ID in donkeys, as it can be valuable for assessing pancreatic beta cell function.

When plasma triglyceride concentrations are very high (opalescent plasma/serum), tissue fatty infiltration of various organs is likely to occur, interfering with their function. Increased liver enzyme activity (GGT, alkaline phosphatase, glutamate dehydrogenase, aspartate aminotransferase, etc.) is common, although this is not specific for hepatic lipidosis (fatty liver) [4]. There could be azotemia secondary to renal dysfunction, dysrhythmias and increased cardiac troponin I activity from myocardial dysfunction, and diarrhea from intestinal fatty infiltration [4]. Pancreatitis due to pancreatic fatty infiltration can be observed with high plasma lipase and amylase activities [16]. Some hyperlipemic donkeys can have impaired glucose dynamics secondary to ID, which could be consequence of pancreatic or liver dysfunction from fatty infiltration, from systemic inflammation, and/or as a result of activation of the hypothalamic–pituitary–adrenal axis. Cortisol interferes with insulin signaling and promotes ID and hyperinsulinemia. Since insulin inhibits adipocyte hormone-sensitive lipase, this further worsens fatty acid mobilization.

Liver echogenicity can be increased due to fatty infiltration and edges can be rounded (enlargement), although this is not specific to this condition. Liver enlargement secondary to fatty infiltration can be severe, causing abdominal pain that in some cases can lead to liver rupture due to higher friability. Depending on the case and potential differential diagnoses, liver biopsy can be considered because it can yield valuable information to differentiate hepatic lipidosis from other conditions and has prognostic value. The benefits and complications of biopsy should be considered because these animals have a higher risk of bleeding from fatty infiltration and abnormalities of coagulation. Histopathology score for horses can be applied to donkeys [17]. Serum biomarkers of liver fibrosis (hyaluronic acid) and an equine-adapted “Enhancer Liver Fibrosis” (eELF) score correlate with liver fibrosis in donkeys, but are less accurate than when used for horses and ponies [17]. 

#### 2.1.7. Treatment

Treatment is based on reducing lipolysis and restoring a positive energy balance. Reducing lipolysis is necessary to address the underlying cause of the negative energy balance (e.g., sepsis, colic, pleuropneumonia, laminitis, etc.), eliminate stressful conditions (lactation, pregnancy, loneliness, etc.) and control clinical signs (e.g., pain, fever, etc.). The approach to restore a positive energy balance will depend on gastrointestinal function (Table 1), the presence of anorexia, systemic health, metabolic status and finances.

The effect of heparin (regular and low molecular weight) on lipoprotein lipase has not been studied in donkeys, although it is used by some clinicians (Table 6) [18]. Lipoprotein lipase activity is maximal in hyperlipemic horses and ponies; thus, exogenous heparin administration is unlikely to further promote triglyceride removal from the circulation [19]. It is important to note that heparin increases the risk of bleeding [20]. These same conclusions could be drawn about the effect of exogenous insulin administration on the activity of this enzyme to remove circulating triglycerides and, at the adipocyte level, to inhibit hormone-sensitive lipase activity and lipolysis, but there is also a risk of insulin-induced laminitis.

#### 2.1.8. Prognosis

The presence of hyperlipemia worsens the prognosis of the primary condition. Mortality rate is high (40–80%) if not addressed rapidly [3]. Triglyceride concentration measurement is highly recommended in any ill donkey, regardless of condition, dullness, behavioral changes (included reluctance to move), inappetence, late pregnancy and lactation or under stressful situations (transport, husbandry, farrier, hospitalization, death of bonded companion, etc.). If a donkey needs to be hospitalized, it is advisable to house them with a companion to reduce stress and facilitate management and diagnostic procedures, but also to reduce the risk of hyperlipemia in the companion.

## 3. Endocrine Diseases

Donkey metabolic syndrome (DMS) and pituitary pars intermedia dysfunction (PPID) are the two most common endocrine disorders diagnosed in donkeys. Other less frequent diseases are thyroid gland abnormalities and disturbances of calcium and phosphorus secondary to parathyroid gland dysfunction and systemic inflammation (e.g., sepsis, colitis, intestinal strangulation, pleuropneumonia, etc.).

### 3.1. Pituitary Pars Intermedia Dysfunction (PPID) 

#### 3.1.1. Introduction

PPID, formerly named Cushing’s disease (due to similarities with canine and human Cushing’s disease), is a common disorder in old donkeys.
***PPID keynotes***Minimal epidemiological data available;Pathogenesis assumed to be like in horses;Beware of hypertrichosis and calm behavior;Resting ACTH in healthy donkeys is higher than in horses;Caution with seasonally adjusted ACTH ranges;TRH-stimulation test recommended for diagnosis;Pergolide dosing like in horses;Measure ACTH at 2–3 months and then 6–12 months.

#### 3.1.2. Epidemiology

Although epidemiological data are scarce, it can be assumed that the prevalence of PPID is higher in donkeys than in horses and ponies due to their longer life expectancy. Based on data from horses, donkeys older than 15 years of age have a higher risk [21]. A breed predisposition has been documented for horses, with Arabian horses and ponies showing higher prevalence [22], but this remains unknown in donkeys. Sex does not seem to have an effect on the development of PPID or severity. A geographical (temperate and climate) effect has been described in horses [23], likely linked to daylight hours, but the same has yet to be elucidated in donkeys.

#### 3.1.3. Pathophysiology

There is no reason to believe that the pathophysiology of PPID in donkeys is different to that in horses and ponies [24]. PPID is a neurodegenerative disease (which explains why age is a predisposing factor), where the dopaminergic inhibition of the pars intermedia melanocytes from hypothalamic neurons is lost. This leads to an increase in the synthesis and subsequent cleavage of proopiomelanocortin (POMC), releasing different peptides (ACTH, α- and β-melanocyte-stimulating hormone [MSH], β-endorphin [β-END], corticotropin-like intermediate lobe [CLIP], etc.) into the bloodstream which are in part responsible for the clinical signs observed in PPID animals. 

#### 3.1.4. Clinical Signs

These are similar to those described for horses and ponies [21], including hypertrichosis, predisposition to infections and parasitism, polydipsia–polyuria, muscle wasting, pendulous abdomen, fat redistribution, recurrent laminitis, insulin dysregulation, reproductive abnormalities, lethargy, orthopedic problems, etc. Due to the calmed temperament of donkeys and the long hair coat of some breeds, PPID signs can go unnoticed for a long time. 

#### 3.1.5. Diagnosis

Diagnosis is based on resting plasma ACTH concentrations. It is important to mention that healthy donkeys have higher resting plasma ACTH concentrations than horses [25,26], and thus, clinicians must be cautious when using equine guidelines [27], which could result in misdiagnosis and false positives depending on the season (Table 2). Similar to horses, a seasonal effect has been also described in donkeys, with higher concentrations in autumn [28], increasing from July to November (Table 2). Moreover, ACTH concentrations in horses and ponies are different between radioimmunoassay (RIA: Millipore, Burlington, MA, USA), chemiluminescent immunoassay (CLIA: Immulite, Siemens, Munich, Germany), electrochemiluminescence immunoassay (ECLIA: Cobas e, Roche diagnostic, Rotkreuz, Switzerland) and immunofluorescent immunoassay (IFIA: AIA 360, Tosoh Bioscience, San Francisco, CA, USA) [29]. Thus, donkey-specific cut-off values adjusted to the season must be established for each analyzer. Meanwhile, the following ranges established using Immulite 1000 can be used: spring, 12.5 pg/mL; summer, 53.2 pg/mL; fall, 77.2 pg/mL; winter, 12.8 pg/mL [28]. It is noteworthy that these studies were carried out in the Northern Hemisphere, and ideally, they should be generated for the Southern Hemisphere (month modification), since ACTH concentrations seem to be influenced by a circadian rhythm associated with daylight hours. 

Sample processing is crucial and can result in false negative results if samples are not collected in EDTA tubes and not centrifugated/separated/frozen rapidly or the shipment is not performed under refrigerated conditions. 

Other peptides released by the pituitary pars intermedia such as α-MSH or β-END are also increased in PPID horses [30]. Alpha-MSH is also influenced by season (higher in autumn months), sample collection and TRH stimulation, but is not affected by stress, transportation, exercise or pain [30]. These peptides have not been evaluated in donkeys. 

In donkeys with clinical signs consistent with PPID but resting ACTH concentrations within normal or non-diagnostic ranges, dynamic testing is recommended (Table 3). Equine protocols including the TRH stimulation and dexamethasone suppression (DST) tests have been evaluated in donkeys, with the former giving more reliable results [31]. A lower protirelin (synthetic TRH) dose (0.5 mg/IV) is used in small and miniature donkeys. In countries where protirelin or chemical-grade TRH are not available, DST can be used, but it is more likely to give equivocal results, mainly in early stages of the disease. Other dynamic tests are no longer recommended.

**Table 2:** Seasonally adjusted resting ACTH concentrations from several donkey studies compared to horse guidelines.

	Ref	Technique	Analyzer		ACTH (pg/mL)			
					December–June	July–November	August	September–October
**Donkeys**	[25]	ND	ND		66.7 ± 20.7 [May–June]	ND	ND	ND
	[32]	CLIA	ND		17.8 (16.5–19.5) [November–June]	37.9 (28.9–36.9) [July–October]		
	[33]	IFIA	Tosoh AIA 360		2.7–30.4 [November–June]	9.0–49.1 [July–October]		
	[26]	CLIA	Immulite 2000 xpi		5–55.4		19.5–143	
	[28]	CLIA	Immulite 2000		17.3 (5–80.5)	40.9 (12.4–214)	88.1 (46–259)	97 (35.7–319)
	[22] *	CLIA	Immulite 1000		35 (22–66) ^a^	63 (40–149.5) ^a^	105 (68–187) ^a^	136 (77–>200) ^a^
	[31] ^#^	CLIA	Immulite 1000		ND	ND	79.2 [65.8]	ND
**Horses**	[27]	CLIA	Immulite 2000 xpi	PPID likely	<15	<15	<20	<30
				Equivocal	15–40	15–50	20–75	30–90
				PPID unlikely	>40	>50	>75	>90

Data are expressed as median (range), median [interquartile range], mean ± standard deviation or minimum–maximum. CLIA, chemiluminescent immunoassay; IFIA, immunofluorescent immunoassay; ND, not described. * Retrospective study of laboratory records (no disease status classification). ^#^ Donkeys suspected of having PPID. ^a^ Data estimated from figures reported by Durham et al. [22].

#### 3.1.6. Treatment

Treatment is similar to that in horses [21]. Pergolide (a dopaminergic agonist type 2) is the preferred pharmacologic agent that has been shown to be effective in donkeys with PPID. Recently, the pharmacokinetics and pharmacodynamics of pergolide were described in donkeys, with faster metabolism and clearance compared to horses [34]. Donkeys have good absorption of pergolide, but also display a cumulative pattern, suggesting that current doses used in horses are appropriate (Table 6). Like horses, some donkeys may fail to respond to pergolide. In these animals, increasing the dose or a combination with cyproheptadine (Table 6) should be considered. There is no pharmacologic information regarding cyproheptadine in donkeys. If anorexia develops, the daily dose should be split between morning and afternoon, reduced, or stopped and resumed 4–5 days later with lower doses and increased progressively. Based on its cumulative kinetics, after days of treatment, perhaps treatment every other day could be a potential option. Cabergoline (another dopamine receptor agonist) has been evaluated and occasionally used in horses refractory to pergolide, but there is no information regarding its use in donkeys.

Since PPID is a chronic, progressive and irreversible disease, life-long treatment is imperative to provide constant dopaminergic melanotrope inhibition. In most donkeys, clinical signs improve with pergolide treatment, including behavior and physical condition (lethargy, haircoat, weight gain and lameness). Follow-up ACTH measurements 3 and 6 months after treatment initiation, and then annually, is recommended. 

#### 3.1.7. Prognosis

Prognosis in donkeys is considered good with proper pharmacologic treatment and regular ACTH measurements. Additional geriatric husbandry care is needed, with regular deworming, vaccinations, dental care and farriery.

### 3.2. Donkey/Asinine Metabolic Syndrome (DMS/AMS)

#### 3.2.1. Introduction

DMS is the most common endocrine disorder in donkeys, where obesity (regional adiposity) and insulin dysregulation (ID) are the main clinical features.
***DMS keynotes***High energy efficiency;Scarce epidemiological data available;Pathogenesis assumed to be like in horses;Functional entero-insular axis;Obesity, ID and HAL are common signs;Minimal information on resting insulin cut-off and ranges;Glucose curves are right-shifted;OST is recommended, but the protocol is not adapted to be donkey-specific;Weight loss is the main treatment, but equine diets are not recommended;Common drugs lack PK/PD studies.

#### 3.2.2. Epidemiology

No donkey-specific epidemiological data are available, but animals of any age, gender and breed can suffer from DMS [1]. Although this syndrome is associated with overfeeding and obesity and is more frequent in developed countries, it also occurs in developing countries with high-quality pastures. The role of vitamin D deficiency (vitamin D_2_ or D_3_) in the pathogenesis of ID in donkeys has not been evaluated [35]. 

#### 3.2.3. Pathophysiology

The mechanisms involved in the pathogenesis of DMS are similar to those in horses [4], although specific studies have not been carried out in donkeys. Briefly, hypertrophy of adipocytes (obesity) leads to the release of proinflammatory cytokines by macrophages residing in the adipose tissue (adipose tissue inflammation). These cytokines decrease glucose uptake by insulin-responsive tissues (peripheral insulin resistance) and stimulate pancreatic β-cells to release insulin. Hyperinsulinemia is also exacerbated by reduced hepatic insulin clearance. Carbohydrate-rich diets (grain; pastures) can also decrease insulin sensitivity. In horses and ponies, hyperinsulinemia alters the behavior of lamellar cells [36,37], resulting in hyperinsulinemia-associated laminitis (HAL) which has been linked to proliferation and epidermal lamellar cell dysfunction due to insulin-like growth factor 1 (IGF-1) receptor activation [38]. Other mechanisms including the direct effect of cytokines on lamellae tissue, reduced perfusion (by endothelin-1), impaired glucose metabolism by lamellar epithelial cells, and the direct effect of weight on the hoof likely contribute. 

It was recently shown that donkeys have a functional entero-insular axis (EIA) [39]. Carbohydrate challenges stimulated a rapid release of glucose-dependent insulinotropic polypeptide (GIP) and active glucagon-like peptide-1 (aGLP-1) in healthy donkeys. An exaggerated EIA response has been proposed to contribute to ID in horses [40]. However, this remains to be determined in donkeys. 

#### 3.2.4. Clinical Signs

Obesity (regional adiposity) and recurrent endocrinopathic laminitis are the main clinical signs [21]. Importantly, laminitis can also be observed in lean donkeys. Infertility and altered lipid metabolism (hyperlipemia) can be also observed [4]. In contrast, the clinical relevance of hypertension (important in human metabolic syndrome) remains to be documented in donkeys. In laminitis-prone ponies on summer pastures and in horses with metabolic syndrome after insulin infusion, it has been observed that there is an increase in blood pressure [41,42].

#### 3.2.5. Diagnosis

- *Insulin dysregulation*

The principles for ID diagnosis in horses apply to donkeys [13]. In addition to physical evaluation, measurements of resting serum/plasma insulin concentrations are crucial in ID diagnosis (Table 4). Donkeys have similar baseline insulin concentrations to horses [25,33,43], with values less than 20 µIU/mL considered normal. Donkey-specific cut-off values for ID diagnosis have not been established, and donkeys with basal not-fasted insulin concentrations higher than 20 µIU/mL need further investigation for ID diagnosis. Insulin concentrations can differ between methods [44]; beware that donkey-specific cut-off values have not been established for each technique/analyzer. A web application to convert insulin concentrations between analyzers has been developed [45].

It is not recommended to measure resting insulin concentrations in fasted donkeys, and thus, providing some hay or straw overnight is suggested. Resting insulin concentration after feeding is good indicator for laminitis risk development in ponies [46]. However, this has not been assessed in donkeys. 

In donkeys with clinical signs compatible with DMS but resting serum insulin concentration in the normal (<20 µIU/mL) or non-diagnostic range (20–50 µIU/mL) according to horse guidelines [47], dynamic testing is recommended (Table 5). Dynamic tests have been characterized in donkeys [39,48]. Glucose disposal is right-shifted in donkeys, suggesting lower intestinal absorption (oral challenges), delayed EIA response, reduced cellular glucose uptake (hepatic or peripheral tissues) or decreased renal clearance. Further studies are needed to elucidate the mechanisms involved. 

The oral sugar test (OST) and oral glucose test (OGTT) are dynamic tests recommended for horses [47]; however, no data on their reliability for ID diagnosis in donkeys are available. The intravenous glucose tolerance test (IVGTT) was more reliable than the combined glucose–insulin test (CGIT) for ID donkeys [49]. Previous studies on the OST and OGT in healthy donkeys showed that glucose disposal is right-shifted (similar to CGIT and IVGTT), and peak glucose was lower compared to that in horses [39]. This indicates that sample timing and cut-off values should be modified, but guidelines for horses are still used in donkeys. For the OST, at least 0.45 mL/kg of corn syrup (Karo^®^ Light corn syrup) is recommended for donkeys by the authors (Table 5). An in-feed OST or the use of other commercial glucose mixtures have not been evaluated in this species. 

**Table 4:** Basal insulin concentrations from several donkey studies compared to horse guidelines.

	Ref	Technique	Analyzer (Manufacturer)	BCS		Insulin (µIU/mL)
**Donkeys**	[25]	ND	ND	ND		1.3 (0–6.6)
	[50,51]	ND	ND	ND		4.9 ± 0.5
	[51]	ND	ND	<3.5/5 *		7.3
				>3.5/5 *		20.9
	[5]	IRMA	KIP1251 (DIASource ImmunoAssays, Louvain-La-Neuve, Belgium)	4–9		9.1 (7.4–14.7)
				4–6		9.9 ± 0.8
				>7		10.3 ± 0.6
	[33]	IFIA	AIA 360 (Tosoh)	ND		0–15.1
	[43]	RIA	Coat-a-Count (Siemens)	2–3.5		2.7 ± 4.6
				4–6		6 ± 4.6
				6.5–9		22.3 ± 4.6
	[26]	CLIA	ADVIA Centaur XPT (Siemens)	ND		0.7–14.4
**Horses**	[47]	CLIA	Immulite 2000 xpi (Siemens)	ND	Non-diagnostic	<30
					ID suspect	30–75
					ID	>75
		RIA/CLIA	ND/Immulite 1000 (Siemens)	ND	Non-diagnostic	<20
					ID suspect	20–50
					ID	>50

Data are expressed as median (range), mean ± standard deviation or minimum–maximum. BCS, body condition score; CLIA, chemiluminescent immunoassay; IFIA, immunofluorescent immunoassay; IRMA, immunoradiometric assay; RIA, radioimmunoassay. * BCS is expressed out of 5. ND, not described.

**Table 5:** Protocols for dynamic testing in donkeys with insulin dysregulation.

Test	OST	OGT	IVGTT	CGIT
*Protocol*	Provide one flake of hay overnight or 6 h prior to sampling. Collect baseline blood sample for resting glucose and insulin determination. ^a,b^			
	Administer 0.45 mL/kg/PO of Karo Light corn syrup	Administer dextrose 1 g/kg (20% solution) through a nasogastric tube	Administer a bolus of 50% dextrose (300 mg/kg/IV)	Administer a bolus of 50% dextrose (150 mg/kg, IV) followed by regular insulin ^b^ (0.1 IU/kg/IV)
	Second blood sample at 60–75 minutes ^c^	Second blood sample at 120–150 minutes ^c^	Second blood sample at 150–180 min	Second blood sample at 60–75 min
*Interpretation*	ID if insulin is higher than 50 µIU/mL ^d^	ID if insulin is higher than 80 µIU/mL ^d^	ID if blood glucose is above baseline ^d^	ID if blood glucose is above baseline ^d^
*Observations*	^a^ Check with the reference laboratory whether plasma or serum is preferred for insulin measurement. Oxalate fluoride tubes are recommended if glucose will be measured later or if the sample will be frozen. Fresh blood can be used for hand-held glucometers. ^b^ Crystalline rapid action insulin. ^c^ Precise blood collection time has not been validated for ID diagnosis in donkeys. ^d^ Insulin cut-off value has not been validated for ID diagnosis in donkeys.			

CGIT: combined glucose–insulin test; ID: insulin dysregulation; IVGTT: intravenous glucose tolerance test; OGT: oral glucose tolerance test; OST: oral sugar test.

Blood biomarkers (cytokines, amino acids and extracellular microvesicles) as well as metabogenomic and lipidomic profiles and their role in the pathogenesis of EMS/ID are being evaluated in horses [52,53]. No data are available for donkeys. The fecal microbiome has been suggested to contribute to ID/EMS in horses [54,55], and while the fecal microbiome has been described in donkeys, an association with DMS remains to be investigated.

- *Obesity*

Obesity is diagnosed based on physical evaluation and using a donkey-specific BCS (>6 out of 9) and neck score (NS, >2 out of 4) [5,56]. Ultrasonography can be useful to assess intra-abdominal and subcutaneous fat [57]. Hepatic enzyme activity can be increased in some obese ID donkeys. This can be explained by hepatic lipidosis inducing a decreased hepatic insulin clearance, or vice versa, an impaired glucose hepatic intake inducing hyperlipemia and hepatic lipidosis. It is important to mention that healthy donkeys have higher GGT activity (up to 80 IU/L) compared to horses [10]. Obese donkeys tend to have higher insulin concentrations and lower insulin sensitivity compared to lean ones [43]. Hyperinsulinemia was recently linked to non-alcoholic fatty liver disease in horses [58].

- *Laminitis*

The diagnosis of laminitis in donkeys is similar to that in horses. However, it is important to use donkey-specific radiographic measurements [59,60].

#### 3.2.6. Treatment

The management of DMS in donkeys is based on reducing body weight, promoting insulin sensitivity and managing laminitis.

- *Obesity*

Weight loss is the cornerstone of DMS management [4]. A progressive diet modification with caloric restriction is crucial for an appropriate weight loss program without risk of hyperlipemia development [61]. The principles of diet management are similar for donkeys and horses. Ideally, diets should have less than 10% of non-structural carbohydrates (NSC), daily dry matter forage consumption should be between 1 and 1.5% of body weight, reduce the number of hours at pasture or consider a grazing muzzle (in some animals, this could increase the risk of hyperlipemia), avoid grazing when plants are growing fast or accumulating sugars, avoid treats (apples, carrots, mints, etc.) and promote physical activity considering orthopedic problems (walker or hand walking, turnout in a pen, etc.). Ideally, hay should be evaluated for nutritional composition and energy content. In addition, soaking the hay for 30–120 min or using a hay steamer to reduce NSC prior to feeding are valid options. Commercial diets to manage obesity in horses are not appropriate for donkeys due to their caloric content and high energy efficiency. Studies on the pharmacology of sodium levothyroxine (Thyro-L^®^) in donkeys have not been conducted , but equine doses are used to increase metabolic rate and promote insulin sensitivity (Table 6) in obese donkeys with laminitis (or other orthopedic problems) or those that are refractory to weight loss measures [4]. Nutraceuticals, such as resveratrol, that have antioxidant properties and increase insulin sensitivity in other species are being used in horses [62], ponies and donkeys, but research information on their use in donkeys is lacking.

- *Insulin dysregulation*

Weight loss can be sufficient to correct ID in most cases. When ID appears in lean donkeys or when weight loss is insufficient, pharmacological treatment should be considered. There are no studies on the pharmacology of metformin in donkeys and current dosing protocols have been extrapolated from horses (Table 6). In donkeys that become anorectic, reducing the dose for a few days and resuming with a lower dose or frequency should be considered. Other drugs used in horses with ID (Table 6), including sodium–glucose transporter type 2 (SGLT-2) inhibitors, incretin analogs or dipeptidyl peptidase-4 inhibitors (DPP-4), have not been evaluated in donkeys; however, they are being used by some clinicians. It is important to note that SGLT-2 inhibitors increase triglyceride concentrations and occasionally liver enzymes. Therefore, before starting treatment, it is important to measure triglyceride concentrations and repeat the measurement few days later to adjust dosing. Other side effects in humans include urinary tract infections secondary to glycosuria, but it has not been yet reported in horses or donkeys.

- *Laminitis*

Equine protocols for laminitis treatment apply to donkeys (analgesia, NSAIDs, acetaminophen, gabapentin, etc.), taking into consideration hoof anatomical differences [60]. An anti-IGF-1 receptor antibody has been evaluated with satisfactory results in horses with induced HAL [63]. Whether this novel therapeutic alternative could be used in laminitic donkeys is still unknown.

#### 3.2.7. Prognosis

In donkeys where ID is addressed only with weight loss and they have mild laminitis (no sinking or rotation), the prognosis is usually good, but in donkeys with severe laminitis, insufficient weight loss and no response to metformin administration, the prognosis is poor. 

### 3.3. Other Endocrine Disturbances

#### 3.3.1. Thyroid Gland Diseases

Donkeys have higher plasma thyroid hormone concentrations (fT3, tT3, rT3, fT4 and tT4) than horses, with younger donkeys showing higher values compared to older ones [64]. No effect of gender has been observed. Drugs such as phenylbutazone or dexamethasone decrease thyroid hormone concentrations in horses, but their effect has not been evaluated in donkeys. Nonetheless, their effects should be taken into consideration prior to thyroid hormone measurements to avoid a misdiagnosis of hypothyroidism [1]. Dynamic tests (TRH stimulation test, TSH stimulation test and T3 suppression test) to assess thyroid function have not been evaluated in donkeys. Thyroid gland adenomas or neoplasia prevalence seem to be lower in donkeys than in horses (authors opinion).

#### 3.3.2. Calcium–Phosphorus Homeostasis Disorders

Nutritional secondary hyperparathyroidism can be seen in donkeys consuming diets rich in phosphorus, poor in calcium or containing oxalate-rich plants [65]. Donkeys have higher ionized calcium and calcitriol, but lower parathyroid hormone (PTH) concentrations than horses [4]. Serum PTH concentrations in donkeys are variable.

Donkeys may develop hypercalcemia, hypophosphatemia and normal-to-low PTH concentrations due to tumors producing PTH-related protein (PTHrP). Healthy animals have very low or undetectable concentrations. This is important to be differentiated from chronic renal failure, where there is also hypercalcemia and hypophosphatemia. In the case of chronic renal failure, creatinine concentrations are often elevated, which is not usually the case in paraneoplastic syndrome in which the neoplasia does not involve the kidneys. Similar to PTHrP, some malignancies may release other endocrine factors (endocrine tumors) with various consequences (paraneoplastic syndrome), but the prevalence is very low [66].

## 4. Conclusions, Further Recommendations and Forthcoming Research

The number of donkeys receiving regular and specialized veterinary care have been increasing in recent years; however, donkey-specific information is limited. Thus, diagnosis and treatment protocols are commonly extrapolated from horses under the false assumption that both species are similar. Clinicians must be aware of species-specific idiosyncrasies to avoid misdiagnosis, unnecessary treatments and increased costs. For example, glucose–insulin disposal differs between donkeys and horses (which affects the interpretation of glucose dynamic testing in the diagnosis of ID), and resting ACTH concentrations are higher in healthy donkeys compared to horses (which invalidates the use of horse ACTH cut-off values for PPID diagnosis).

Future research should be focused on enhancing our understanding of disorders of donkeys, their epidemiology, diagnostic protocols and therapies. Donkey-specific cut-off values and ranges for insulin and ACTH need to be further developed and validated. In addition, seasonal variations should be generated for commonly used analyzers and techniques (Immulite versus AIA, IRMA versus CLIA, etc.). The standardization and simplification of dynamic testing is also important for the diagnosis of ID and ACTH. Pharmacokinetic/pharmacodynamic studies of drugs commonly used to treat metabolic and endocrine diseases (metformin, levothyroxine, SGLT-2 inhibitors, etc.) are necessary to improve outcomes. Finally, advances in the microbiome and metabolome description of horses with ID and PPID could serve as a blueprint for future research in donkeys.

## Figures and Tables

**Table 1:** Approach to restore a positive energy balance in hyperlipemic donkeys.

- Donkeys with appetite and a functional gastrointestinal (GI) tract: o Offer different palatable feedstuffs. o Provide treats such as apples, carrots, mints, molasses, corn syrup, etc. - Anorectic donkeys with a functional GI tract: o Enteral nutrition: ▪ Administer commercial enteral formulations through a small-bore nasogastric tube (cost can be a limitation). ▪ Administer a homemade blended gruel: Dissolve 250 g of pelleted alfalfa in 2–5 L of plain warm water. Administer 1–3 L (250 kg BW) every 2–4 h through the nasogastric tube. Dextrose (100–300 g), sodium bicarbonate, potassium chloride, magnesium sulfate and sodium phosphate can also be added to the mixture. In donkeys younger than 6 months of age, a milk replacer or starter grain can be added. One inconvenience with enteral nutrition could be tube obstructions if the mixture is too tick. Diarrhea or soft feces can be observed secondary to excessive water administration or intestinal dysbiosis. o Oral honey administration: 20–30 mL every 4–6 h. o Daily vitamin and mineral supplementation. - Donkeys with non-functional GI: gastric reflux, ileus, etc. o Partial parenteral nutrition (without lipids): ▪ Start with 2–5% dextrose/glucose continuous infusion (CRI). Tip: Use 5 L of lactated Ringer’s solution and 500 mL of 40–50% glucose at 2–3 mL/kg/h. ▪ This solution can be used for several days, although depending on the primary condition (e.g., colitis), amino acids must be added. ▪ Determine blood glucose levels hourly for 4 h: ➢ If hyperglycemia is observed (>160 mg/dL), decrease the rate. ➢ If hyperglycemia persists (sepsis, DMS/ID): ˟ Regular insulin (fast action, Table 6). ˟ Protamine zinc insulin (Table 6). ˟ Continue checking glucose (hypoglycemia could develop). ˟ Although the effect of persistent hyperinsulinemia has not been evaluated in donkeys at this moment, it is assumed that it will also cause laminitis, similar to in horses.

**Table 3:** Protocols for dynamic testing in donkeys with pituitary pars intermedia dysfunction.

Test	TRH Stimulation Test	DST *
*Protocol*	Fasting has no influence. Collect at any time of the day. Avoid sampling acutely laminitic, stressed or ill animals, or after exercise or sedation with α2-agonists. Collect baseline blood sample for resting ACTH ^a^ (TRH) or cortisol ^b^ (DST) determination.	
	Administer protirelin (synthetic TRH) or chemical-grade TRH: 0.5–1 mg/IV.	Administer dexamethasone: 40 µg/kg/IM.
	Second blood sample at 10 min.	Second blood sample at 15–19 h.
*Interpretation*	PPID if ACTH is higher than 110 pg/mL.	PPID if cortisol is higher than 1 µg/mL.
*Observations*	^a^ EDTA tubes are preferable for ACTH; however; check with the laboratory for collection instructions. Rapidly centrifuge and freeze plasma. Ship under refrigeration. ^b^ Check with the reference laboratory whether plasma or serum is preferred for measurement.	

* False negatives can be observed, only recommended when protirelin is not available. DST, dexamethasone suppression test; TRH, thyrotropin-releasing hormone.

**Table 6:** Common drugs used for treatment of metabolic and endocrine disturbances in donkeys.

Drugs	Dose	Route	Interval	Observations
**Heparins**				
Dalteparin	50–100 IU/kg	SC	24 h	Increases LPL activity
Enoxaparin	40–80 IU/kg	SC	24 h	Increases LPL activity
Heparin sulfate	70 IU/kg	IV-SC	12–24 h	Aggregates erythrocytes and increases LPL activity
**Insulins**				
Regular insulin	0.05–01 IU/kg	IV-IM	6 h or CRI	Increases LPL activity and inhibits HSL
Protamine zinc insulin	20–60 IU	IM	24 h	Increases LPL activity and inhibits HSL
**Synthetic thyroxine (tT_4_)**				
Sodium levothyroxine	0.05–0.1 mg/kg	PO	24 h	Improves insulin sensitivity
**Dopamine receptor agonist type 2**				
Pergolide	0.002–0.01 mg/kg	PO	24 h	Anorexia, lethargy and oral ulcers
**Anti-H_1_ receptor, anticholinergic (M) and antiserotonergic (5-HT)**				
Cyproheptadine	0.2–0.6 mg/kg	PO	12–24 h	An appetite stimulant
**Biguanides**				
Metformin	15–50 mg/kg	PO	8–12 h	Anorexia; does not induce hypoglycemia
**Sodium–glucose transporter inhibitor type 2 (SGLT-2)**				
Canagliflozin	0.3–0.6 mg/kg	PO	24 h	Increases blood triglycerides
Ertugliflozin	0.03–0.05 mg/kg	PO	24 h	Increases blood triglycerides
Velagliflozin	0.3 mg/kg	PO	24 h	Increases blood triglycerides
**Dipeptidyl peptidase-4 inhibitor (DPP-4)**				
Sitagliptin	1.5 mg/kg	PO	24 h	

CRI, constant rate infusion; HSL, hormone-sensitive lipase; LPL, lipoprotein lipase.

## Data Availability

No new data were created or analyzed in this study. Data sharing is not applicable to this article.
